# The Effect of Casting Technique and Severe Straining on the Microstructure, Electrical Conductivity, Mechanical Properties and Thermal Stability of the Al–1.7 wt.% Fe Alloy

**DOI:** 10.3390/ma16083067

**Published:** 2023-04-13

**Authors:** Andrey Medvedev, Olga Zhukova, Nariman Enikeev, Vil Kazykhanov, Victor Timofeev, Maxim Murashkin

**Affiliations:** 1 Institute of Physics of Advanced Materials, Ufa University of Science and Technology, 32 Zaki Validi Str., 450076 Ufa, Russia; 2 Laboratory of Dynamics and Extreme Characteristics of Promising Nanostructured Materials, Saint-Petersburg State University, 198504 Saint Petersburg, Russia; 3 Department of Electrical Engineering, Siberian Federal University, 79 Svobodnyy Prospekt, 660041 Krasnoyarsk, Russia

**Keywords:** Al–Fe alloys, electromagnetic mold, severe plastic deformation, equal channel angular pressing, cold rolling, ultrafine grain structure, mechanical properties, electrical conductivity, thermal stability

## Abstract

This paper features the changes in microstructure and properties of an Al–Fe alloy produced by casting with different solidification rates followed by severe plastic deformation and rolling. Particularly, different states of the as-cast Al–1.7 wt.% Fe alloy, obtained by conventional casting into a graphite mold (CC) and continuous casting into an electromagnetic mold (EMC), as well as after equal-channel angular pressing and subsequent cold rolling, were studied. Due to crystallization during casting into a graphite mold, particles of the Al_6_Fe phase are predominantly formed in the cast alloy, while casting into an electromagnetic mold leads to the formation of a mixture of particles, predominantly of the Al_2_Fe phase. The implementation of the two-stage processing by equal-channel angular pressing and cold rolling through the subsequent development of the ultrafine-grained structures ensured the achievement of the tensile strength and electrical conductivity of 257 MPa and 53.3% IACS in the CC alloy and 298 MPa and 51.3% IACS in the EMC alloy, respectively. Additional cold rolling led to a further reduction in grain size and refinement of particles in the second phase, making it possible to maintain a high level of strength after annealing at 230 °C for 1 h. The combination of high mechanical strength, electrical conductivity, and thermal stability can make these Al–Fe alloys a promising conductor material in addition to the commercial Al–Mg–Si and Al–Zr systems, depending on the evaluation of engineering cost and efficiency in industrial production.

## 1. Introduction

The electrical industry is one of the leading aluminum consumers. At present, aluminum is widely used as a conductor material in the cable industry and electrical machines, in the on-board wiring of cars and various aircraft, in lighting engineering, and in the production of electrical installation work [1,2,3]. While yielding to copper in electrical conductivity, aluminum surpasses it in many other parameters. For example, the weight and price per ton of aluminum wires are 52% less than the weight and price of copper wires [4,5]. Therefore, in recent years, much attention has been paid to the improvement of technologies and methods for obtaining and processing aluminum alloys for electrical purposes [6].

The main disadvantage of aluminum alloys for electrical purposes is their low strength and heat resistance. In recent decades, successful attempts have been made to improve these characteristics while maintaining high electrical conductivity, primarily through adding the transition and rare earth elements [7,8], as well as using various methods of deformation heat treatment [9,10,11].

Much attention is currently being paid to the development of methods for obtaining and processing aluminum-based alloys, ensuring the formation of an ultrafine-grained (UFG) structure in them [12]. Studies have shown that it can provide an improvement in the complex of physical and mechanical properties in a number of alloys in comparison with analogues produced using traditional technologies [13,14,15].

An effective approach for the formation of UFG is severe plastic deformation (SPD). In particular, the most common methods are high-pressure torsion (HPT) [16,17] and equal-channel angular pressing (ECAP) [18,19]. The ECAP method is especially attractive in that its continuous modification, ECAP-Conform, makes it possible to obtain long-sized products [20].

At present, for the manufacture of aluminum wires and cables, aluminum alloys of the 8000 series (8030 and 8176 in particular) are widely used [21,22]. These alloys contain iron in small proportions, which significantly changes the properties of aluminum. The main problems with the aluminum alloy grades 8030 and 8176 are their relatively low mechanical strength and thermal stability. Earlier studies have demonstrated that SPD can significantly enhance the mechanical strength of Al–Fe alloys while retaining high electrical conductivity [16]. Additional opportunities to control the properties of cast Al alloys are associated with their production technique, such as the casting method. Aluminum alloy grades 8030 and 8176 are manufactured by continuous casting and rolling or a combined continuous casting and rolling-pressing process. Recently, electromagnetic casting (EMC) has become more widespread for casting aluminum alloy ingots. The main advantage of the EMC method is the absence of contact between the molten metal and the mold walls, which improves the surface quality of the ingot and the casting speed by 10–30% and provides a particularly high cooling rate of 10^3^–10^4^ K/s [23,24]. A combination of these two methods could provide a level of mechanical and electrical properties unattainable by any other technique or their combination.

It is known that the original cast structure containing dispersed particles in the nanometer size range can be obtained in aluminum alloys by continuous casting in an electromagnetic mold due to the high crystallization rate. Previously, the authors showed that this approach works for aluminum alloys containing 0.5 and 2.5% Fe, which upon casting were subjected to two-stage deformation treatments, including ECAP and CR [25]. Despite the rather high level of strength achieved in the alloy with 2.5% iron and a UFG structure, its electrical conductivity was low due to the high content of the second-phase particles of the Al_2_Fe type. On the contrary, in the 0.5% iron alloy, the low strength does not allow us to recommend this alloy for further use.

In this work, a study was carried out aimed at finding a rational iron content in an aluminum alloy, which is necessary to achieve a combination of maximum strength, electrical conductivity, and heat resistance. Due to the formulation of this problem, an alloy with an intermediate iron concentration between 0.5% and 2.5%, namely 1.7%, was chosen for achieving this goal. To compare the structural characteristics of the electromagnetically cast Al–1.7Fe alloy, as well as the physical and mechanical properties, an alloy of a similar chemical composition, but obtained by conventional casting into a graphite mold, was used in the work.

## 2. Materials and Methods

Samples of Al–1.7 wt.% Fe alloy (hereinafter Al–1.7Fe alloy) obtained by different casting methods were used as research materials. The chemical composition of the alloys is presented in Table 1.

Part of the Al–1.7Fe alloy samples in the form of rods with a diameter of 22 mm and a length of about 200 mm was made by conventional casting into a graphite mold. The cooling rate of the billet during the implementation of this casting method was about 20 K/s. The alloy was prepared on the basis of primary aluminum grade A99. Melting was carried out in an GRAFICARBO GF 1100 electric resistance furnace (Graficarbo S.R.L., Zorlesco, Italy) in a graphite crucible at 820–850 °C at Russia’s National University of Science and Technology “MISIS” (Moscow, Russia).

The other part of the alloy samples was obtained in the form of rods with a diameter of 11 mm and a length of 2 m by continuous casting into an electromagnetic mold (EMC) at the LLC Scientific and Practical Center for Magnetic Hydrodynamics (Krasnoyarsk, Russia). The alloy was prepared on the basis of primary A85 aluminum with the addition of a Fe80Al20 master alloy in the required proportion. Before casting, the melt temperature exceeded 800 °C. The casting speed in the EMC was about 12.5 mm/s.

Previous studies have shown [23,24] that the method of continuous casting in EMC makes it possible to obtain a microstructure in blanks of aluminum alloys alloyed with transition and rare earth elements, containing particles of intermetallic compounds homogeneously distributed in the aluminum matrix, with sizes close to the nanometer range. The high crystallization rate, which, as a rule, is 10^3^–10^4^ K/s when casting in EMC, also provides an unusually high concentration of alloying elements of transition metals, such as Zr and/or Mn, in the aluminum solid solution [26].

From cast samples of alloys obtained by both methods, samples of square section with a size of 10 × 10 × 80 mm were made using a wire-cutting APTA-120 machine (Delta-Test, Fryazino, Russia). They were subjected to a two-stage deformation treatment. At the first stage, the samples were subjected to 4 passes of ECAP at room temperature (RT). ECAP was carried out in a rig with an angle of conjugation of the channels of 120 degrees, along the route Bc [20,27]. Such or very close ECAP conditions are usually used to form a UFG structure in low-alloy aluminum alloys [27,28]. The selected channel conjugation angle in the tooling coincides with the geometric parameters of the tool used in the implementation of the continuous ECAP-Conform method [20].

At the second stage, the samples treated with ECAP were subjected to cold rolling (CR). It was carried out on a laboratory 2-roll reversing mill for six technological transitions. The total degree of the rolling deformation was 85%. As a result of rolling, thin strips 1.1 mm thick were obtained.

To determine the level of heat resistance, some of the samples after two-stage processing were annealed in a Nabertherm B180 (Nabertherm, Liliethal, Germany) electric furnace at temperatures of 230 °C and 280 °C for 1 h in accordance with the requirements of the international standard IEC 62004:2007 [29].

The microstructure was studied by transmission electron microscopy (TEM) using a JEOL JEM 2100 (JEOL, Tokyo, Japan) microscope at an accelerating voltage of 200 kV. Objects for studying the microstructure were electropolished by two-jet polishing of thin foils on a Struers Tenupol-5 unit (Struers, Copenhagen, Denmark) using a solution of 20% nitric acid and 80% methanol. Polishing was carried out at a solution temperature of −20 °C and a voltage of 20 kV. To obtain statistically reliable results, at least three foils per state were studied. The microstructure was also studied using scanning electron microscopy (SEM) on a JSM-6490LV microscope (JEOL, Tokyo, Japan) at an accelerating voltage of 15 kV. Element mapping was performed by the EDX method using an INCA X-Act attachment to an electron microscope. ImageJ software (ver. 0.4.0.) was used for image processing and quantitative measurements.

X-ray diffraction (XRD) analysis was conducted with a Bruker D2 Phaser (Bruker, Billerica, MA, USA) diffractometer using CuKα radiation. The values of the lattice parameter, *a*, the coherent domain size, *D*, and the elastic microdistortion level, <ε^2^>^1/2^, were calculated by the Rietveld refinement method using MAUD software (ver. 2.992). To calculate dislocation density (*ρ*), Equation (1) was used in the same way as in [30].

(1) $$\rho =\frac{2\sqrt{3}{\left(\varepsilon^{2}\right)}^{1/2}}{D\times b}$$

where *b* is the Burgers vector value and *D* is the coherent domain size.

Mechanical tests were carried out at room temperature with a strain rate of 10^−3^ s^−1^ on an Instron 5982 (Instron, Norwood, MA, USA) universal testing machine. Yield stress, σ_0.2_, ultimate tensile stress, σ_UTS_, and elongation to failure δ of the samples were determined by the results of stretching the samples with the dimensions of the working part (2.0 × 1.0 × 6.0 mm). To obtain reliable results, at least three samples were tested for each experimental point.

IACS = ω_Al_/ω_Cu_ × 100%, (2)

where ω_Al_ is the measured electrical conductivity of aluminum alloy and ω_Cu_ is the conductivity of annealed chemically pure copper (58 MS/m).

## 3. Results

### 3.1. Microstructure of Al–1.7Fe Alloys in the Initial State and After Cold Deformation

**Figure 1:**
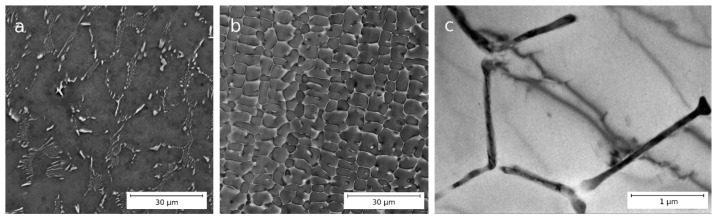
Microstructure of alloys CC (**a**) and EMC (**b**) in their initial states, SEM; (**c**) TEM micrograph of the EMC sample.

Figure 1 shows the microstructure of Al–1.7Fe alloys in the conventional cast (hereinafter CC) state and the state obtained by casting into an electromagnetic mold (hereinafter EMC). In both alloys, the structure is an aluminum matrix with inclusions of the second phase Al_x_Fe_y_ of crystallization origin. A more detailed examination shows that the inclusions of the second phase have the form of thin, parallel plates or needles. Despite the same iron content, alloy CC is characterized by larger and more rarely located particles of intermetallic compounds. Thus, the average thickness of intermetallic plates in alloy CC is 350 ± 130 nm, and in the alloy obtained by EMC, the thickness of the plates is in the nanometer size range of −90 ± 20 nm. Figure 1a also displays several intermetallic particles (with sizes up to 2 microns) that are coarser than the major fraction of particles in the specimen’s volume. However, their number density is rather low, and the presence of such large intermetallic particles in Al–Fe alloys, even in larger amounts, does not have any significant effect on the properties of the SPD-processed samples [30]. Hence, these particles have been excluded from consideration when estimating the mean particle dimensions. The volume fraction of intermetallic particles in the CC alloy is 6.1 ± 1.0%, and in the EMC alloy, it is 4.9 ± 1.0%. The size of the dendritic cell in the CC alloy is 13.0 ± 5.0 µm, and in the EMC alloy, it is 4.6 ± 1.5 µm. The much smaller size of dendritic cells and intermetallic particles in EMC alloy samples is explained by a higher crystallization rate during casting.

**Figure 2:**
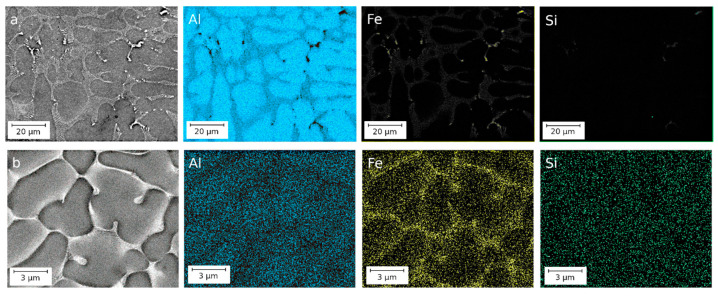
Elemental mapping of the microstructure of the CC (**a**) and EMC (**b**) alloys, SEM.

Energy-dispersive X-ray spectroscopy, carried out with precision from particles of the second phase and an aluminum matrix, made it possible to establish that in all alloys iron is absent in the matrix (Figure 2).

**Figure 3:**
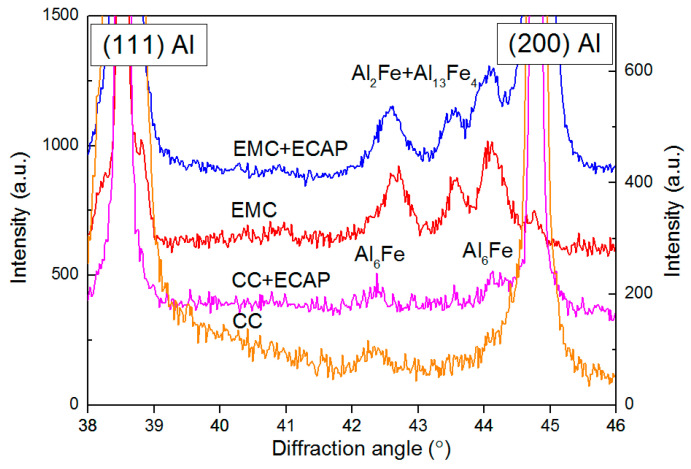
XRD profiles of the CC and EMC Al–1.7Fe alloys in the as-cast states and after ECAP.

Figure 3 and Table 2 show the results of X-ray diffraction analysis of CC and EMC alloys in their initial states and after ECAP. According to the analysis of the X-ray profiles (Figure 3), in the initial state in the CC alloy, peaks from the metastable Al_6_Fe phase are observed; its presence was confirmed in this system earlier [31]. Carrying out ECAP on alloy CC does not lead to a change in its phase composition.

Figure 3 shows XRD data for the CC and EMC alloys. In contrast to alloy CC, in EMC alloy iron is predominantly associated with a phase of the Al_2_Fe type, with inclusions of the Al_13_Fe_4_ phase. The Al_2_Fe phase is rarely observed and is mainly formed in alloys with nonequilibrium crystallization conditions [25]. Nevertheless, the particle morphology in the CC and EMC alloys, as mentioned above, is similar (except for the particle size), so that the type of phases does not affect their morphology. The lattice parameter of aluminum in the EMC alloy after ECAP does not change, which also indicates the absence of signs of the formation of a solid solution. Alloys EMC and CC after ECAP are characterized by similar X-ray diffraction parameters, such as the dislocation density. It is also interesting to note that, despite the difference in phase composition, the morphology of intermetallic particles remains the same for the alloys under study.

**Figure 4:**
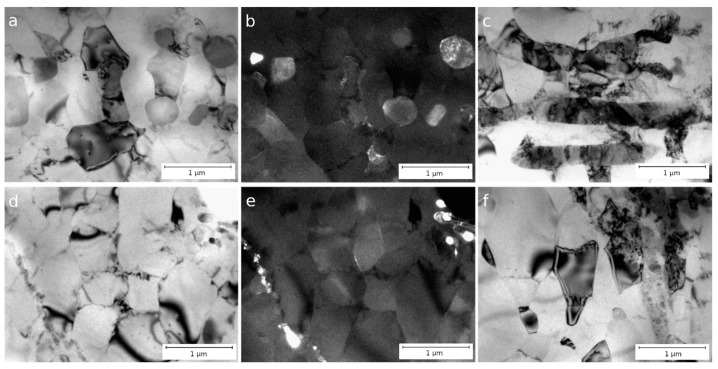
Microstructure of Al–1.7Fe alloys after ECAP: (**a**–**c**) CC and (**d**–**f**) EMC, longitudinal section, TEM.

Figure 4 shows the microstructure of alloys CC (a, b) and EMC (c, d) after ECAP in a longitudinal section. The study of the microstructure of the alloys using TEM showed that after four passes of ECAP, both equiaxed and elongated grains were found. These grains had a clearly defined preferred orientation (metallographically) and were oriented in accordance with the deformation direction implemented in the ECAP process.

Due to severe plastic deformation, partial fragmentation of intermetallic particles occurs in both alloys. Nevertheless, the thickness of intermetallic plates in alloys after ECAP remains practically unchanged—in alloy CC, the average particle size after ECAP changed from 370 ± 130 nm to 334 ± 65 nm, and in alloy EMC, from 90 ± 20 to 94 ± 24 nm (Table 3). The distribution of particles in both alloys remains uneven, and particle accumulations are mainly located along the original intermetallic plates (Figure 4). A decrease in the average grain size occurs during ECAP—the average grain size of the CC alloy after ECAP is 694 ± 159 nm, and that of the EMC alloy is 620 ± 20 nm. Individual particles and arrays of fragmented particles act as barriers to the migration of grain boundaries.

**Figure 5:**
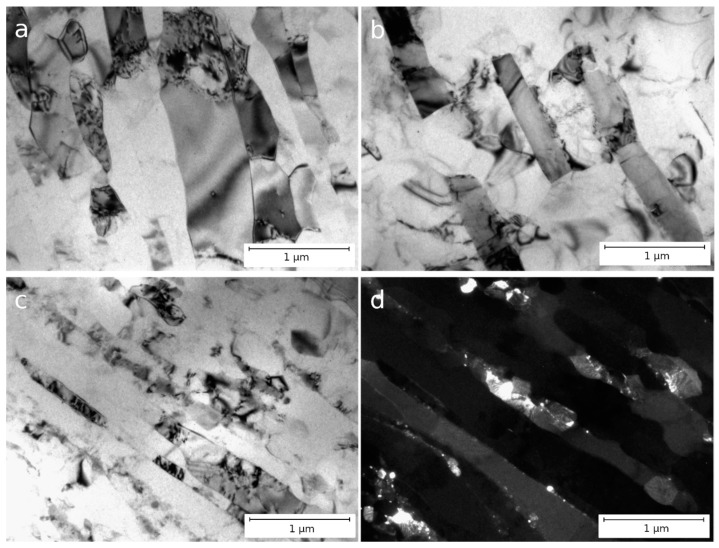
Microstructure of Al–1.7Fe alloys after ECAP and subsequent CR with a reduction ratio of 85%: (**a**,**b**) CC and (**c**,**d**) EMC, longitudinal section, TEM.

Figure 5 shows the microstructure of alloys CC (a, b) and EMC (c, d) after ECAP and subsequent cold rolling with a reduction ratio of 85%. Electron microscopic analysis of alloy CC by TEM showed the presence of grains elongated along the direction of deformation. Quantitative analysis showed that the average grain width is 256 ± 100 nm (with a maximum grain width of up to 450 nm) and that the grain length reaches several microns. In addition, even cold rolling did not lead to particle refinement, and plates of initial particles (with an average width of 264 ± 40 nm) are still observed in the alloy structure (Figure 5a,b). ECAP and subsequent rolling also caused serious changes in the microstructure of the EMC alloy (Figure 5c,d). In the plane of the longitudinal section, a pronounced fibrous UFG structure is observed with an average fiber (grain) width of 290 ± 20 nm, the length of which is a micron or more. Lamellar borders are straight and clear. The structure contains both large intermetallic inclusions that have not undergone changes from the cast state and a fraction of small intermetallic particles with an average size of 70 ± 50 nm. (Figure 5d, Table 3).

### 3.2. Mechanical Properties and Electrical Conductivity

Table 4 shows the measurement data for the physical and mechanical properties of alloys CC and EMC in their initial states. The EMC alloy is distinguished by a lower level of electrical conductivity and a considerably higher level of σ_0.2_ and σ_UTS_ compared to the CC alloy. At the same time, the plasticity of the EMC alloy is two times higher than that of the CC alloy. The difference is in the different fraction of intermetallic particles in the alloys—small intermetallic plates in the EMC alloy act as an effective barrier to electrons, reducing the electrical conductivity of the alloy.

An analysis of the physical and mechanical properties of CC and EMC alloys after ECAP and CR is presented in Table 4. ECAP in CC and EMC alloys leads to the expected increase in the ultimate strength in both alloys (up to 201 MPa in the CC alloy and up to 268 MPa in the EMC alloy) and to a slight decrease in electrical conductivity in the EMC alloy (up to 48.5% IACS). The electrical conductivity of the alloy CC does not undergo noticeable changes. The ductility of the alloys levels off and reaches a level of 10–12%. In general, such changes in properties due to ECAP are due to the grinding of intermetallic particles due to their fragmentation and the formation of a UFG structure formed by a network of predominantly high-angle grain boundaries as well as a high density of lattice dislocations (Table 2). According to the X-ray diffraction data, ECAP does not lead to the formation of a solid solution (the aluminum lattice period does not change), which means that its effect on the properties can be neglected.

Cold rolling, carried out after ECAP, continues the trend of strengthening alloys—the ultimate strength of alloy CC increases to 257 MPa, and that of alloy EMC increases up to 298 MPa. Simultaneously, the electrical conductivity (up to 53.3% IACS in the CC alloy and up to 51.3% IACS in the EMC alloy) and ductility (up to 15.9% in the CC alloy and up to 17.1% in the EMC alloy) of the alloys under study also increase.

### 3.3. Thermal Stability of UFG Al–1.7Fe Alloys

Thermal stability is a key factor when using aluminum-based materials at elevated temperatures, as aluminum alloys are prone to active recovery processes. Poor thermal stability leads to a rapid decrease in mechanical strength upon heating, and the purpose of thermal stability testing is to determine the service temperature at which the reduction in mechanical strength will be acceptable. Thermal stability tests of aluminum alloys are carried out according to IEC 62004:2007 [29].

**Figure 6:**
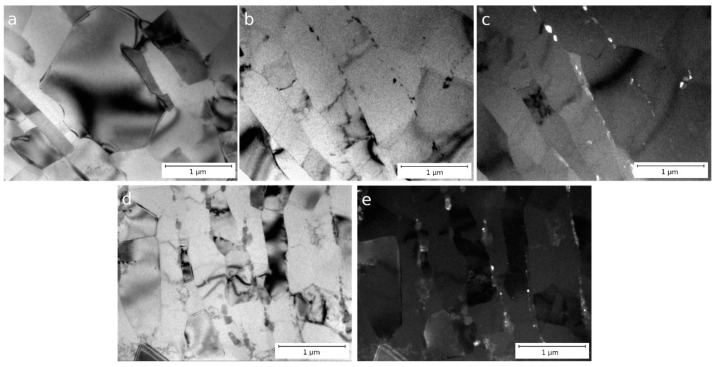
Microstructure of Al–1.7Fe alloys after ECAP, CR, and subsequent annealing at 280 °C for 1 h: (**a**–**c**) CC and (**d**,**e**) EMC, longitudinal section, TEM.

The TEM images of samples of alloys CC and EMC after ECAP, CR, and subsequent annealing at 280 °C for 1 h are shown in Figure 6. In alloy C, an increase in grain width up to 685 ± 262 nm is observed while maintaining the overall grain elongation in the rolling direction. In alloy C, just as in the state after rolling, large particles of the intermetallic phase are observed. At the same time, accumulations of small intermetallic particles are clearly visible along the grain boundaries (Figure 6a–c), the average size of which is 77 ± 18 nm.

The microstructure of the EMC alloy is in many respects similar to the microstructure of the CC alloy, except that the intermetallic phase is crushed and no large intermetallic inclusions are observed in this state. In the EMC alloy, grains remain elongated in the rolling direction, although after annealing, the average grain width increases to 575 ± 25 nm (Table 3). The presence of intermetallic particles with an average size of 50 ± 20 nm, distributed along the grain boundaries and more densely located than in alloy CC, could be the reason for the stability of the grain in the annealed sample—annealing at a temperature of 280 °C could lead to a significant grain growth in a similar aluminum alloy [11].

Alloys CC and EMC after ECAP and CR demonstrate the stability of their properties after annealing at a temperature of 230 °C for 1 h (Table 5). According to [29], this means that these alloys can operate at 150 °C for a long time without loss of performance. Annealing at 280 °C leads to a significant reduction in mechanical strength to an unacceptable level in both deformed alloys. The highest level of strength after ECAP, CR, and annealing at 280 °C is observed in the EMC alloy—235 MPa. The electrical conductivity of the alloy in this state is 55.3% IACS.

In both alloys, after annealing at 230 °C, a decrease in plasticity is observed. If for the EMC alloy it is relatively small and lies within the error value, then in the CC alloy the drop is more significant—from 15.9% to 9.3%. It is quite probable that the reason for this behavior is due to the recovery process that has taken place and the decrease in the dislocation density required to ensure plastic flow. The presence of large, coarse particles also adversely affects the ductility of the CC alloy. On the contrary, annealing at 280 °C leads to a quite expected increase in ductility in both alloys.

**Figure 7:**
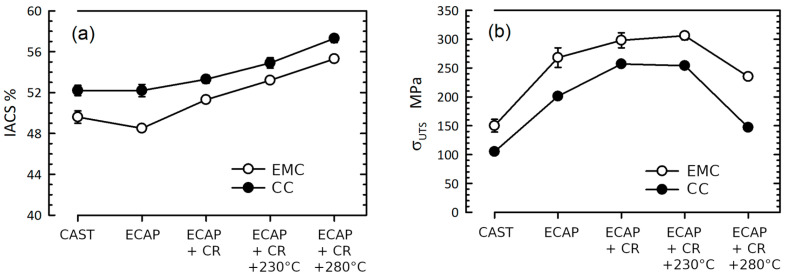
Evolution of the properties of Al–1.7Fe alloys: (**a**) electrical conductivity, (**b**) tensile strength.

The results of a study of the electrical conductivity and strength characteristics of aluminum alloys in their initial states after casting and casting in EMC, after processing by the ECAP method and additional HP, as well as annealing at temperatures of 230 °C and 280 °C, are presented in Figure 7.

## 4. Discussion

### 4.1. Microstructural Basis for Electricity-Strength Relationship Evolutionary Behavior

Despite the fact that the behavior of the mechanical and electrical properties of the studied alloys during ECAP and subsequent cold rolling was predictable—an increase in strength with a simultaneous decrease in electrical conductivity—the studied alloys differed in the degree of change in these properties.

Since the chemical composition of the alloys is identical, the reason for the difference in properties due to identical deformation processing is related to a different method of obtaining as-cast materials. The casting technique parameters have provided considerable differences in the qualitative and quantitative phase compositions of the alloys. According to Figure 3, the intermetallic phase in the CC and EMC alloys consists of fundamentally different phases of the Al_X_Fe_Y_ type—predominantly Al_2_Fe with the inclusions of the Al_13_Fe_4_ phase in the EMC alloy and the Al_6_Fe phase in the CC alloy. Analysis of the SEM images showed that the intermetallic phase in the EMC alloy is located much more densely than in the alloy CC, despite the similar volume fraction of the intermetallic phase. This is a direct consequence of the high cooling rate of the EMC alloy during crystallization, which provides an increased number of crystal nucleation centers. The following deformation treatments do not result in changing the type of the pre-existing Al–Fe phase and can mostly result in the fragmentation of intermetallic particles.

To determine whether the morphology or type of the intermetallic phase is the determining factor in the change in the physical and mechanical properties of alloys as a result of cold deformation, it is necessary to estimate the contributions of various structural mechanisms.

Table 6 shows the contributions of structural mechanisms to the mechanical and electrical properties of CC and EMC alloys after ECAP. These calculations are based on the bottom line of the XRD data and the results of the statistical analysis of the TEM data—the average grain size and the average particle size [32,33,34]. The ECAP state was chosen for the structural mechanism impact calculation because the effect of the strength increase is most prominent in this state. Such a notable change in properties will reduce the error in the calculations. In addition, CR after ECAP leads to the redistribution of the particles, making them uneven and less susceptible to the evaluation.

It should be noted that this calculation model has its limitations. Thus, the calculation is carried out for equiaxed grains and particles close in shape to spherical and uniformly distributed in the volume of the material [35].

Note that we did not take into account the solid solution effects that could possibly be induced by different casting cooling rates and SPD. In principle, acceleration of the cooling rate during casting is capable of forming supersaturated solid solution states, as demonstrated for the low-soluble Al–Co system [36]. The solubility of iron in aluminum under normal conditions is almost negligible (0.025 wt.% according to [37]). Moreover, the cooling rate is not expected to influence the solubility of iron in aluminum [37]. SPD under extreme conditions can induce dissolution in the Al–Fe system [38,39], which was shown to be accompanied by a noticeable change in the alloy’s lattice parameter [38]. According to XRD data, no significant changes in the lattice parameter occur after both casting and SPD, which indicates the absence of a solid solution in the alloys in the studied structural states.

According to Table 6, the largest of the calculated contributions to the yield strength and electrical resistance is the contribution of the grain boundaries. The contribution of the dislocation density is insignificant, and the contribution of the solid solution is close to zero. Thus, the unaccounted factor is the contribution from dispersed particles; however, it is not possible to calculate it due to the non-uniform distribution of particles in the bulk of the material. Despite this, one can try to qualitatively estimate the contribution of dispersed particles to the strength and electrical conductivity of alloys.

The volume fraction of the intermetallic phase in CC and EMC alloys is comparable. Accordingly, the difference in the calculated and experimental values of strength and electrical conductivity (Δ_exp−calc_) will depend on the size and shape of particles, their distribution density, and the type of particles themselves.

Analysis of the microstructure showed that in the state after ECAP, the CC and EMC alloys contain non-fractured particles of the intermetallic phase (Figure 4). The size of these particles is comparable to the size of the as-cast intermetallic particles for each alloy. At the same time, the EMC state is characterized, in addition to the presence of large particles, by the presence of a fraction of small (less than 100 nm) particles formed as a result of fragmentation of plates in the intermetallic phase. Thus, while the morphology of the particles in alloy CC remained almost unchanged and did not entail significant changes in electrical conductivity, the formation of a fraction of nanosized particles in the EMC alloy led to a decrease in electrical conductivity. In the case of a large difference in the average particle size, it would be more appropriate to use the particle distribution density parameter to assess their influence on the properties of the material.

The distribution density of particles can be represented as the average distance between particles. Different models for calculating the contribution of dispersed particles to the yield stress of alloys somehow consider this indicator. Regardless of the calculation model, an increase in the average distance between particles by a factor of two, other things being equal, leads to a decrease in the contribution of this structural mechanism by about two times; that is, the dependence is linear. The value of Δ_exp−calc_ for strength and electrical conductivity in alloys CC and EMC differs by about 1.5 times, which generally corresponds to the difference in the distribution density of intermetallic particles (Table 6).

As regards the types of phases, it should be noted that the intermetallic particles in the CC alloy are represented by the Al_6_Fe phase, and in the EMC alloy, by the Al_13_Fe_4_ and Al_2_Fe phases. The Al_6_Fe and Al_13_Fe_4_ phases are orthorhombic and non-coherent to aluminum, while the Al_2_Fe phase is orthorhombic and semi-coherent to aluminum. It is quite probable that the semi-coherence of the Al_2_Fe phase leads to its interaction with conduction electrons and dislocations, which differs from the Al_6_Fe and Al_13_Fe_4_ phases.

### 4.2. Combination of Strength and Electrical Conductivity

One of the distinguishing features of this work, in addition to the different quantitative and qualitative phase compositions, is the reduced effect of the strength-conductivity tradeoff for aluminum alloys.

If the strength behaves quite predictably, then the electrical conductivity either does not change (during deformation) or increases (after annealing) (Figure 7). This deviation from the standard choice between increased strength and ductility makes it possible in the future to realize alloys capable of hardening without loss of electrical conductivity.

**Figure 8:**
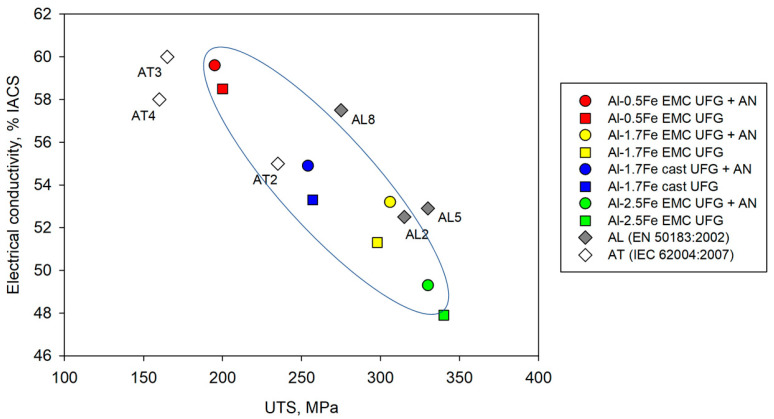
Mechanical strength and electrical conductivity of the alloys obtained in this study (Al–1.7Fe alloys) and in a previous work (Al–0.5Fe and Al–2.5Fe) [25], in comparison with the data taken from IEC 62004:2007 [29] and EN 50183:2002 [40] requirements.

Figure 8 presents data on the strength and electrical conductivity of alloys in the Al–Fe system. Figure 8 presents the results of the Al–1.7Fe alloys after the ECAP and CR study, as well as the results of previous studies regarding Al–0.5Fe and Al–2.5 alloys obtained by EMC and subjected to ECAP and CR [25]. The analysis of this data allows one to state that the Al–Fe alloys with an iron content of up to 2 wt.% in the UFG state are characterized by an outstanding combination of electrical conductivity and mechanical strength. Particularly, the combination of strength and electrical conductivity achieved by Al–1.7Fe alloys as a result of severe plastic deformation allows them to compete with heat-resistant alloys in the Al–Zr system of the AT series traditionally used in electrical engineering [29] and even with the strongest thermally hardened alloys in the 6101 and 6201 Al–Mg–Si systems [40].

## 5. Conclusions

1. It is shown that the casting method affects the structure and properties of aluminum alloys. The EMC method allows, firstly, to obtain smaller particles of the intermetallic phase (to change the quantitative phase composition), and secondly, to change the type of crystallizing intermetallic compounds (the qualitative phase composition).
2. The formation of the Al_2_Fe phase in this system was confirmed, which was previously shown for Al–0.5Fe and Al–2.5Fe alloys. This phase is metastable, and, as the analysis of the contributions of microstructural mechanisms to strength and electrical conductivity showed, its presence, together with the changed morphology of intermetallic particles, leads to an increase in the strength of the alloy without loss of electrical conductivity during ECAP and subsequent CR.
3. ECAP and subsequent CR lead to the expected increase in the strength of the alloys under study, and the increase is more pronounced in the EMC alloy due to the initially finer fraction of intermetallic particles and their crystal structure. The more unexpected change was the non-decrease and even increase in electrical conductivity in these alloys, which allows us to talk about overcoming the concept of finding a balance between strength and electrical conductivity in aluminum alloys.
4. Alloys have shown high thermal stability—up to 230 °C for 1 hour, which is equivalent to operating at 150 °C for a theoretically unlimited time. The data obtained make it possible to recommend these alloys as thermally stable conductor materials in addition to commercial Al–Zr and Al–Mg–Si systems.

## Figures and Tables

**Table 1:** Chemical composition of Al–1.7Fe alloys (wt.%).

Casting Method	Al	Fe	Si	Σ (Zn, Ti, Ni, V, Cu)
Conventional casting (CC)	Rem.	1.71	0.12	<0.04
Electromagnetic casting (EMC)	Rem.	1.65	0.03	<0.04

**Table 2:** Results of the XRD studies of the CC and EMC Al–1.7Fe alloys in the as-cast state and after ECAP.

State		*D_XRD_*, nm	<ε^2^>^1/2^, %	*a*, Å	*ρ*_dis_ × 10^13^, m^−2^
CC	Cast	-	-	4.0493	-
	ECAP	330 ± 9	0.0035 ± 0.0006	4.0500	1.3
EMC	Cast	-	-	4.0499	-
	ECAP	200 ± 5	0.0043 ± 0.0010	4.0498	2.6

**Table 3:** Average grain and particle size in Al–1.7Fe alloys.

	Initial (Dendritic Cell Size)		ECAP		ECAP + CR (Grain Width)		ECAP + CR + AN (Grain Width)	
Casting type	CC	EMC	CC	EMC	CC	EMC	CC	EMC
Grain size, nm	13 ± 5 µm	4.6 ± 1.5 µm	694 ± 159	620 ± 20	256 ± 100	290 ± 20	685 ± 252	575 ± 25
Particle size, nm	370 ± 130	90 ± 20	334 ± 65	94 ± 24	264 ± 40	70 ± 50	77 ± 18	50 ± 21

**Table 4:** Physical and mechanical properties of CC and EMC alloys after ECAP and CR.

State	Alloy	ω, MS/m	IACS, %	σ_0.2_, MPa	σ_UTS_, MPa	δ, %
Cast	CC	30.36 ± 0.26	52.2 ± 0.5	40 ± 7	105 ± 4	18.3 ± 0.5
	EMC	28.77 ± 0.21	49.6 ± 0.6	60 ± 6	150 ± 11	28.8 ± 2.1
ECAP	CC	30.27 ± 0.34	52.2 ± 0.6	179 ± 8	201 ± 4	9.6 ± 0.2
	EMC	28.10 ± 0.40	48.5 ± 0.2	251 ± 8	268 ± 17	11.7 ± 1.6
ECAP + CR	CC	30.96 ± 0.19	53.3 ± 0.4	220 ± 4	257 ± 1	15.9 ± 1.3
	EMC	29.77 ± 0.19	51.3 ± 0.1	276 ± 11	298 ± 13	17.1 ± 1.6

**Table 5:** Physical and mechanical properties of the UFG CC and EMC alloys after thermal stability tests.

State	Alloy	ω, MS/m	IACS, %	σ_0.2_, MPa	σ_UTS_, MPa	δ, %
ECAP + CR + 230 °C, 1 h	C	31.88 ± 0.29	54.9 ± 0.6	190 ± 3	254 ± 1	9.3 ± 0.2
	EMC	30.86 ± 0.20	53.2 ± 0.1	284 ± 8	306 ± 7	14.4 ± 1.4
ECAP + CR + 280 °C, 1 h	C	33.24 ± 0.21	57.3 ± 0.4	126 ± 3	147 ± 1	31.5 ± 2.2
	EMC	32.10 ± 0.14	55.3 ± 0.1	228 ± 2	235 ± 3	17.9 ± 0.6

**Table 6:** Contribution of structural mechanisms to the strength and electrical conductivity of CC and EMC alloys.

Yield Strength, MPa	σ_0_	σ_GB_	σ_disl_	σ_disp_	σ_0.2calc_	σ_0.2exp_	Δ_exp-calc_
CC ECAP	10	48	9	n\a	67	179	112
EMC ECAP	10	51	12	n\a	73	251	178
**Electrical Resistivity, mΩ × m**	**ρ_Alpure_**	**ρ_GB_**	**ρ_disl_**	**ρ_SS_**	**ρ_calc_**	**ρ_exp_**	**Δ_exp-calc_**
CC ECAP	0.02655	0.002248	0.0000004	0	0.02880	0.03304	0.00424
EMC ECAP	0.02655	0.002516	0.0000007	0	0.02907	0.03559	0.00652

Where: σ_0_—alloy lattice resistance to dislocation migration (Peierls-Nabarro stress); σ_GB_—grain boundary hardening; σ_SS_—solid solution hardening; σ_disp_—dispersion (precipitation) hardening; σ_disl_—dislocation hardening. For the second part of the table: ρ_0_—alloy lattice resistance to electron migration; ρ_GB_—grain boundary contribution; ρ_SS_—solid solution contribution; ρ_disl_—dislocation contribution.

## Data Availability

The data presented in this study are available on the reasonable request from the corresponding author.
